# Salivary β2-microglobulin levels in patients with erosive oral lichen planus and squamous cell carcinoma

**DOI:** 10.1186/s13104-020-05135-w

**Published:** 2020-06-17

**Authors:** Fatemeh Nosratzehi, Tahereh Nosratzehi, Ebrahim Alijani, Soha Saberi Rad

**Affiliations:** 1 Department of Chemistry, Faculty of Science, University of Birjand, Birjand, Southern Khorasan Iran; 2 Department of Oral Medicine, Oral and Dental Disease Research Center, Zahedan University of Medical Sciences, Zahedan, Iran; 3 Department of Immunology, School of Medicine, Clinical Immunology Research Center, Zahedan University of Medical Sciences, Zahedan, Iran; 4 Dental Research Center and Department of Oral Medicine, School of Dentistry, Zahedan University of Medical Sciences, Zahedan, Iran

**Keywords:** Salivary β2-microglobulin, SCC, OLP

## Abstract

**Objectives:**

β2 microglobulin, as a biomarker, is used for the diagnosis of oral malignant and pre-malignant lesions. The components of the microglobulin system can directly or indirectly help grow and develop tumors. The present study aims to compare beta-2 microglobulin levels in patients with lichen planus of the esophagus, oral squamous cell carcinoma, and healthy individuals. Further, it evaluated the salivary β2-microglobulin level in malignant and pre-malignant lesions. Oral lichen planus (OLP) is a chronic skin-mucus disorder. Of the total 75 patients referred to Oral Medicine at Dentistry School of Zahedan University of Medical Sciences, 25 were healthy and 25 had oral lichen planus (OLP) and the rest had squamous cell carcinoma (SCC). To collect the saliva samples, unstimulated spitting was used. They were collected between 9 and 12 a.m. Salivary beta2 microglobulin was recorded based on the factory instructions by ELISA optical density method with 450 nm wavelength for each sample. The data were analyzed using descriptive, Kruskal–Wallis and Mann–Whitney and Pearson’s correlation coefficient (SPSS 21).

**Results:**

The salivary β2 microglobulin level in patients with squamous cell carcinoma (SCC) and oral lichen plan (OLP) is significantly higher than that in healthy group. Thus, this index is used for assessing early malignant transformation and oral pre-malignant lesion.

## Introduction

Lichen planus is a common chronic inflammatory mucocutaneous disease and 50% of cases often have oral mucosa. The etiopathogenesis of oral lichen planus (OLP) has yet been poorly understood, but T cell-mediated immunity and inflammatory pathways play a part in its pathogenesis [1]. Many studies refer to autoimmune properties of OLP such as chronicity of the disease, prevalence in adulthood, an inclination to the females. involvement with other autoimmune diseases, an increase in immunosuppressive activity in patients with OLP, and the presence of auto-toxicity cells in OL lesions support the autoimmunity role of disease pathogenesis [2]. Considering that squamous cell carcinomas (SCC) have been developed from LP, several studies have focused on malignant transformation of OLP lesions to oral SCC (OSCC), as it has become a concerning global topic. Based on the definition of WHO, the term “oral lichen planus” is known as a potentially premalignant condition. The molecular mechanisms underlying the development of oral cancer are not clearly known in patients with OLP, but OLP lesions can evolve from normal epithelium or precancerous lesions and the disruption of basement membrane may trigger the Keratinocyte (KC) apoptosis [3].

OSCCis the most common neoplasia of the oral cavity and a serious worldwide health problem; thus, understanding the SCC biomarkers is essential for early diagnosis, better prognosis and the prevention of disease recurrence and a good way to decrease the mortality of patients. Malignant transformation of oral mucosa causes the proliferation of cells, abnormal keratinization, epithelial dysplasia, increased cell motility, and angiogenesis due to gene mutation in cell growth and its regulation. Cancer occurs through genetic changes that cause deregulation of protein, poor cell division, and tissue differentiation, invasion, and metastasis [4].

Tumor indicators have recently been recognized for the early diagnosis of malignancy. In oral cavity carcinomas, different serum indicators including oncofetal proteins (alpha photo protein CEA), B proteins and enzymes (LDH) have been studied. One of the most significant indicators is β2 microglobulin, a protein with light (low weight) molecules (11,800 kDa).It is found on each surface of cell except for erythrocytes which are considered as a light unchangeable chain of compatible histologic antigens [5]. It is abundant in monocytes and lymphocytes [6]. In the normal physiologic state, some amounts of β2 microglobulin can be secreted to the cell or serum due to the intracellular release and it is often extracted from the blood by kidneys [7–11]. Thus, β2 concentration of microglobulin (β2M) is measured by the amount of production and secretion to serum and extraction by kidneys [12]. β2M concentration increases as a result of the kidney’s dysfunction and cells’ turnover [13]. Thus, in individuals with healthy kidneys, an increase in the β2M amount indicates the proliferation of the changed cells. Increasing β2M amount in serum was observed in some pathologic cases including kidney diseases, immunity deficiency, and autoimmune disease. Besides, there was a high level of β2M in some solid and hematologic cancers in the time of diagnosis [14, 15]. Saliva-based analysis has been proposed in recent years and the potentially abnormal markers of oral cavity appear in saliva directly or indirectly. Therefore, its application as a diagnostic fluid can be of special significance. Saliva is a diagnostic tool for assessing markers. It is advantageous because it is cheap for monitoring, safe for collecting, non-invasive, convenient, simple and reproducible, without causing discomfort to the patient [16–18].

Baliah et al. determined the β2M level in serum in patients with oral leukoplakia, oral submucous fibrosis and oral squamous cell carcinoma; and compared to the control group. A total of 100 cases were classified in four groups: the first group contained patients with oral leukoplakia based on the clinical and histopathological reports; the second group consists of patients with oral submucous fibrosis; the third group includes patients with oral squamous cell carcinoma (OSCC) and the last one was the control group. Results have indicated that the mean level of β2M in the serum of the leukoplakia, oral submucous fibrosis, OSCC patients and in the control group was 2597 ± 148.6, 2187.68 ± 678.6, 3166.04 ± 357.7, and 1542.60 ± 377.70 ng/mL, respectively. There was a significant increase in the mean level of β2M concentration in the first and the third groups compared to the control group. However, an increase in β2M concentration in patients with oral submucous fibrosis was not statistically significant. The present study has supported the hypothesis of using β2M concentration as an indicator in patients with oral leukoplakia and oral squamous cell carcinoma [19].

Diwan et al. studied the role of β2M as a tumor indicator in OSCC and leukoplakia patients. For this purpose, OSCC patients (n = 30), leukoplakia patients (n = 23), and normal individuals (n = 20) in the control group were analyzed. Using the logistic regression model, the effect of age and gender was removed from samples due to their influence on β2M concentration. Results showed that β2M concentration was higher in OSCC and leukoplakia patients compared to the control group. Thus, β2 microglobulin in serum can be used as an indicator in the diagnosis of these diseases. Increasing the concentration of β2 microglobulin was positively correlated with grading the histology of OSCC [20].

The study of Gonazales et al. aimed to assess the evidence on the ability to transform to malignant OLR, OLL, and different variables with the highest effect on the development of disease were determined [21]. They investigated 82 studies on a total of 26,742 patients by November 2018. The malignancy speed was reported OLR: 1.72, OLL: 1.88, OLP: 1.14. The analysis of subgroups showed that the ability to be transformed into malignancy depends on variables such as Epithelial dysplasia, the location of the lesion, smoking, consuming alcohol, type of lichen planus and accompanying lichen planus with hepatitis C.Giuliani et al. confirmed that OLP, OLL may be considered a potentially malignant disorder [22].

Thus, considering the high prevalence of oral cancers and oral lichen planus in Zahedan, the lack of similar study, and proving the safety and usefulness ofsaliva as a diagnostic method of oral cancer and Lichen planus, we have analyzed the β2M concentration in these patients.

## Main text

### Materials and methods

In this cross-sectional study, a group of patients including 25 patients with clinical lesions of OLP (bilateral lesions, popular and reticular lesions, and Wickham lines) and, if necessary, with histological confirmation (characterized by a band-like inflammatory infiltrate cells, limited to the surface area of the connective tissue; predominantly mature lymphocytes, accompanied by vascular degeneration of the basal layer of the epithelium); with no other oral lesions (group A), 25 patients with new SCC and reports of pathologist proving SCC, with no oral lesions (group B), and 25 individuals with no history of SCC or OLP lack of systemic disease and medication (group C). Patients who took corticosteroid or any kind of immunosuppressive drug for at least 1 week before the sampling, those who had hematopoietic stem cell transplantation, hepatitis C, lupus erythematosus, Sjogren’s syndrome, graft versus host disease, those who drink alcoholic beverages or smoke, pregnant women and patient with Gingivitis were excluded from the study. The participants signed the consent form before participating in the research proposal. Then, the intraoral examination was done by the specialist of oral diseases under a good unit light, a biopsy of the given area was done and handed to the department of pathology. The results were recorded in the data form. To collect the saliva samples in different groups and control group, unstimulated spitting was used. The samples were collected between 9 and 12 a.m. based on the pre-published protocol. The participants were asked to wash their mouth before spitting in certain tubes and sit upright 5 min after washing and spit in a 50 mL falcon. The samples were sent to the lab shortly after being collected and centrifuged at 2600 round at 4 °C for 15 min, proteinase inhibitor including 10 NL aprotinin (10 mg/mL), 10 NL phenylmethanesulfonyl fluoride (10 mg/mL and 3 NL Sodium orthovanadate (400,000 M, Na2) was added to each milliliter of a floating solution to prevent deregulation of protein. All of the samples were maintained at 80 °C and salivary β2-microglobulin level was recorded according to the instruction of the factory using ELISA kits and BOSTER biological made in France with a sensitivity of 95% and optical density at a wavelength of 450 nm. OSCC and OLP patients received treatment and follow-up.

The data were analyzed using descriptive, Kruskal–Wallis and Mann–Whitney and Pearson’s correlation coefficient (SPSS 21).

### Result

In this study, the data collected from 75 participants were analyzed in three groups of 25 patients (healthy control, patients with OSCC, and patients with OLP). The results of this analysis are listed in subsequent paragraphs.

According to the data in Table 1, Kruskal–Wallis test suggested a significant difference among these three group Post-hoc Tukey test-pairwise comparison- suggested that β2-microglobulin in healthy group was significantly less than that in Oral lichen planus and Oral Squamous Cell Carcinoma groups (P = 0.042); while it was the same in both groups of patients (P = 0.997) (Table 2). In case of β2-microglobulin presence, the predictive value of the positive test, negative test and efficiency of the test was 100%.

**Table 1:** The mean and standard deviation (SD) of β2-microglobulin (mg/L) in participants of each group (healthy controls, SCC, OLP)

Group	Mean	SD	Min	Max
Control	0.6918	0.21934	0.09	0.97
OLP	1.4408	1.41455	0.25	5.45
SCC	1.3729	1.31760	0.28	4.93

**Table 2:** LSD test for comparing the groups in pairs considering the level of β2-microglobulin (mg/L)

I	J	Mean difference (I − J)	p-value	95% confidence level	
				Low limit	High limit
Control	OLP	− 0.74898	0.042	− 1.5501	0.0522
Control	SCC	− 0.6810	0.048	− 1.4900	0.1278
OLP	SCC	0.06788	0.977	− 0.7153	0.8511

### Discussion

In many studies, saliva has been used as a diagnostic medium by dentists and physicians. A decrease and increase in cytokines and inflammatory mediators’ level with different diagnostic factors in saliva make sampling possible which can be done by the patient himself. Some experts considered saliva and it’s derivate a reflection of the physiologic and pathologic changes in the body [23, 24].

β2 microglobulin is a kind of protein with 11,800 kDa molecular weight synthesized by cells containing a nucleus. Its normal amount in the saliva is 2.06 ± 0.36 mg/L [25]. The present study was performed to analyze the level of β2M in the saliva of OSCC and oral lichen planus (OLP) patients. The results have indicated that β2M concentration in the control group was significantly less (0.691 ± 0.21 mg/L) than patients with OSCC (1.1 ± 44.41 mg/L) and OLP (1.1 ± 37.31 mg/L). The amount of β2M in the saliva of these patients is the same.

The study of RupkarPratic et al. indicated that serum level of β2M is a valuable diagnosis factor in the OLP and OSCC patients [26]. The results of their study are completely consistent with those of the present study.

Baliah et al. reported that serum beta-2 microglobulin in patients with oral squamous cell carcinoma was significantly more than that in healthy people. Their study confirmed that beta-2 microglobulin can be applied as an indicator of the tumor for oral squamous cell carcinoma [19].

Various studies Diwan et al. [20], Agrawai et al. [27], Nosratzehi et al. [16] and Singh et al. [18]), suggested that beta-2 microglobulin is considered as a sensitive and specific indicator of oral squamous cell carcinoma.

Kadam et al. has indicated that the serum level of these biomarkers has increased during the development of the disease from the first to the fourth step. These biomarkers are efficient for oral cancer [25].

Viashali and Tupkari have shown a significant relationship between β2M serum level and histological grading of SCC and introduced this biomarker as a sensitive test for diagnosis, analysis, and prognosis as well [28]. Some studies indicated different results. In this regard, Rasool et al. have suggested that the β2M blood level is a better indicator compared to its saliva level [29]. Though, in the present study, the β2M saliva level was significantly higher in patients rather than in normal people. This contradiction could be related to the small sample size, the intensity of the disease, and the patient’s age and sex. Thus, it is essential to determine the differentiated degree of the disease in measuring β2M saliva in metastatic patients.

Because the saliva is a non-invasive, cheap, simple and duplicable tool, it can be used as a diagnostic marker. It potentially analyzed the premalignant and malignant complications. The present study showed that the β2M level in normal people is significantly lower than that in OLP and OSCC patients; β2M concentration in patients with those diseases was the same.

Thus, the results of the present study suggest that these markers are useful for assessing early malignant change, the accuracy of clinical diagnosis and the invasiveness of the cancer of the oral cavity.

## Limitations

It is suggested to perform the studies on larger samples considering different age groups and genders. It is recommended to measure the β2M level in different grades and stages of the oral squamous cell carcinoma.

## Data Availability

Data are available upon request from the corresponding author.

## Abbreviation

β2M: β2-Microglobulin
OLP: Oral lichen planus
SCC: Squamous cell carcinoma
OSCC: OLP lesions to oral SCC
